# Research progress of quercetin on anti-anxiety and anti-depression

**DOI:** 10.3389/fphar.2025.1621510

**Published:** 2025-05-30

**Authors:** Teng Teng, Chunhong Song

**Affiliations:** Shandong Key Laboratory of Traditional Chinese Medicine and Stress Injury, Department of Laboratory Animal Center, Central Hospital Affiliated to Shandong First Medical University, Jinan, Shandong, China

**Keywords:** quercetin, anti-anxiety, anti-depression, mechanism of action, native compound

## Abstract

Anxiety and depression represent two of the most prevalent mental disorders worldwide, with current pharmacotherapies often limited by adverse effects and treatment resistance. Consequently, research into natural products for mental health interventions has attracted growing scientific interest. Quercetin, a bioactive flavonoid ubiquitously present in plant-derived flowers, fruits, and dietary sources, exhibits diverse pharmacological properties, including antioxidant, anxiolytic, and antidepressant activities. This review systematically summarizes the fundamental characteristics of quercetin, focusing on its core molecular mechanisms in alleviating anxiety- and depression-like behaviors, preclinical evidence from experimental models, and the current landscape of clinical investigations. By critically analyzing its therapeutic advantages, translational challenges, and emerging research priorities, this synthesis aims to offer a theoretical foundation for the development of quercetin-based formulations and their clinical implementation in mood disorder management.

## 1 Introduction

Anxiety disorders primarily include panic disorder and generalized anxiety disorder. In recent years, the escalating prevalence of anxiety disorders has exerted a profound impact on human wellbeing, thereby attracting extensive attention across diverse societal sectors. The Global Burden of Disease Study 2019 (GBD 2019) revealed that, influenced by the COVID-19 pandemic, the global prevalence of anxiety disorders reached approximately 374 million individuals in 2020, with a notably higher incidence among females than males (Collaborators, 2021). This mental condition is shaped by a complex interplay of external environmental, physiological, and genetic factors. Patients often experience unfounded fear and anxiety, devoid of specific content, frequently accompanied by symptoms of muscle tension (Pluess et al., 2009). Depression, conversely, is characterized as an emotional dysregulation disorder. Its cardinal symptoms encompass a pervasive loss of interest, physical fatigue, and the emergence of persistent negative emotions. Additionally, patients often display self - negation, and in severe cases, may engage in extreme behaviors such as suicide and self-harm, imposing substantial negative consequences on both society and families (Jellinger, 2024).

Both anxiety and depression are classified as neurotic disorders, sharing overlapping etiologies and pathophysiological mechanisms. Key factors include dysregulation of neurotransmitter systems (e.g., serotonin, dopamine) and the hypothalamic-pituitary-adrenal (HPA) axis, which collectively contribute to the development of these mood disorders. Notably, many patients exhibit comorbid anxiety and depression, and these emotional states frequently co-occur with other medical conditions, such as coronary heart disease, chronic heart failure, hypertension, and certain gastrointestinal disorders. For such patients, comprehensive treatment requires not only management of the primary diseases but also administration of anxiolytic and antidepressant medications. Examples of commonly used therapies include selective serotonin reuptake inhibitors (SSRIs) for depression, such as sertraline or escitalopram, the adverse effects of Escitalopram and Sertraline include nausea, vomiting, dry mouth, and constipation (Sanchez et al., 2014). The benzodiazepine anxiolytics for anxiety, such as lorazepam or clonazepam. For clonazepam, long-term use may lead to physical dependence and tolerance (Dokkedal-Silva et al., 2019). These pharmacological interventions, when combined with primary disease management, aim to address both psychological and physical comorbidities holistically.

Traditional pharmacotherapies, including selective serotonin reuptake inhibitors (SSRIs) and benzodiazepines, remain the mainstay of treatment (DeGeorge et al., 2022). However, their clinical application is hampered by limitations such as delayed therapeutic onset, adverse effects, and the development of treatment resistance. Wang conducted a retrospective cohort study that included 3,688 individuals aged 60 years and older from a depression screening study. A 5-year follow-up found that compared with patients who also had severe depression but did not use SSRIs, those who used SSRIs had a significantly higher risk of developing dementia during the follow-up period (Wang et al., 2016). Similarly, the incidence of hip fractures in individuals treated with the medication was more than twice that in those who did not use antidepressants (Brannstrom et al., 2019). Regarding benzodiazepines, the most serious issue is the tolerance and dependence caused by long-term use. A study on adults found that users of benzodiazepines continued long-term use for more than 7 years after the start of treatment (Rosenqvist et al., 2024). Consequently, the exploration of safe and efficacious novel antipsychotic agents derived from natural products has emerged as a burgeoning research frontier.

Quercetin, also named meletin, is a polyphenolic flavonoid abundantly present in various plant-derived sources, including grapes, broccoli, citrus fruits, onions, and tomatoes. This bioactive compound exhibits a broad spectrum of pharmacological activities, such as antioxidant, anti-inflammatory, anti-allergic, antiviral, immunomodulatory, and anticancer properties (Chiang et al., 2023; Wang et al., 2023; Carrillo-Martinez et al., 2024). In recent years, with the in-depth exploration of its pharmacological actions and mechanisms, preclinical evidence from animal models has continuously validated its therapeutic potential. Notably, quercetin has demonstrated significant neuroprotective effects against neurodegenerative disorders, including Alzheimer’s disease, Parkinson’s disease, and Huntington’s disease (de Oliveira Vian et al., 2024; Pei et al., 2025; Makhdoomi et al., 2024).

Characterized by its molecular formula C15H10O7 and a chemical structure rich in phenolic hydroxyl groups, quercetin exerts antioxidant, anti-inflammatory, and multi-target regulatory functions (Singh et al., 2021), which aligns well with the current trends in drug development. Upon oral administration, the small intestine serves as the primary site for the metabolic transformation of quercetin and other flavonoids, while the remaining fraction undergoes microbial metabolism in the colon. Modulating the intestinal absorption and metabolism processes may thus represent a promising strategy for optimizing the biological effects of quercetin (Murota and Terao, 2003).

## 2 Quercetin modulates neurotransmitters and their receptors to exert anti-anxiety and anti-depressant effects

Samad found that under stress stimulation, the metabolic level of 5-hydroxytryptamine (5-HT) in the stressed state was significantly higher than that in the non-stressed group. Quercetin, endowed with neuroprotective properties, effectively mitigated stress-induced anxiety-and depression-like behaviors in murine models. The compound also counteracted stress-induced lipid peroxidation and the inhibition of antioxidant enzymes. Beyond its impact on 5-HT neurotransmission, quercetin modulated cholinergic neurotransmission, thereby influencing anxiety, depression, and memory functions in mice (Samad et al., 2018).

Gamma-aminobutyric acid (GABA) and glutamate (Glu), essential amino acid neurotransmitters in the central nervous system, play pivotal roles in neural modulation. Reduced GABA levels in the human brain are commonly associated with anxiety manifestations. The GABAA receptor, a ligand-gated ion channel, has been identified in multiple studies as a key regulator of anxiety (Yu et al., 2024). Animal experiments have shown that quercetin exhibits anxiolytic effects comparable to diazepam and exerts a synergistic action, potentially through interaction with the α2 subunit of the GABAA receptor (Tripathi et al., 2024).

The HPA axis is a fundamental component of the neuroendocrine system. Upon stress, the hypothalamus releases corticotropin-releasing factor (CRF) (Domin and Smialowska, 2024). A strong association exists between HPA axis dysregulation and anxiety/depression (Li et al., 2024). Bhutada employed the forced swimming test and social interaction assays, observing that quercetin administration decreased immobility time and increased social interaction time in experimental animals (Bhutada et al., 2010). Quercetin exerted its anxiolytic effects by reducing brain CRF expression, likely mediated directly or indirectly by CRF receptors.

In a mouse model of mild traumatic brain injury (mTBI), the activity of the HPA axis in response to stress was examined. Measurement of corticosterone levels in mouse serum revealed that mTBI induction increased HPA axis activity in mice, and quercetin was able to reduce corticosterone level expression. The anxiolytic effect of quercetin may be related to its ability to normalize the function of the HPA axis by regulating corticosterone levels in animals. (Kosari-Nasab et al., 2019). Behavioral experiments further revealed that quercetin reduced depression-like behaviors in BRAS rats by inhibiting HPA axis excitability, primarily through suppressing the expression level of α2δ-1 and disrupting its interaction with N-methyl-D-aspartate receptors (NMDARs) (Wang et al., 2024).

## 3 Quercetin modulates the immune system to exert an anti-anxiety effect

Quercetin administration remarkably increases the time spent and the number of entries in the open arms of the Elevated Plus Maze (EPM) test, along with the frequency of center zone crossings in the open field test, effectively ameliorating anxiety-like behaviors. These beneficial effects were observed in a mouse model established by lateral ventricle lipopolysaccharide (LPS) injection, which mimics anxiety- and depression-like phenotypes. Mechanistically, the anti-anxiety action of quercetin is closely associated with the inhibition of interleukin-6 (IL-6) and interleukin-1β (IL-1β) expression in the hippocampus (Lee et al., 2020).

Furthermore, studies have shown that methamphetamine abuse can trigger anxiety-like behaviors. In this context, quercetin treatment not only alleviates these anxiety manifestations but also rectifies mitochondrial dysfunction, potently suppresses astrocyte activation, and decreases the levels of pro-inflammatory cytokines, such as IL-1β and tumor necrosis factor-α (TNF-α), ultimately leading to a significant reduction in anxiety behaviors (Chen et al., 2022).

## 4 Quercetin exerts antidepressant effects by modulating oxidative stress

Oxidative stress occurs when the body is exposed to harmful stimuli, resulting in the overproduction of reactive oxygen species (ROS) and other reactive molecular species. Oxidative stress stands as a primary factor in neurodegenerative diseases, affecting not only conditions such as Parkinson’s disease, amyotrophic lateral sclerosis, Alzheimer’s disease, and Huntington’s disease (Teleanu et al., 2022), but also playing a role in the pathophysiology of depression (Bhatt et al., 2020). Elucidating its mechanisms and associated oxidases is crucial for understanding anxiety disorders. Elevated ROS levels trigger peroxidation of cell membrane lipids, leading to the generation of malondialdehyde (MDA). Elevated MDA levels have indeed been detected in the serum of depressed patients (Wong et al., 2022).

In an olfactory bulbectomy (OB) mouse model of depression, quercetin demonstrated antidepressant effects in classic behavioral assays, including the open field test, forced swimming test, and tail suspension test. OB-induced depression was associated with decreased hippocampal glutathione (GSH) levels, increased superoxide dismutase (SOD) levels, and elevated lipid hydroperoxide (LOOH) levels. Notably, quercetin selectively reversed the increase in LOOH levels, suggesting that its antioxidant properties contribute to the alleviation of depressive symptoms (Holzmann et al., 2015).

Furthermore, in a PC12 cell model challenged with amyloid-β peptide 25–35 (Aβ25-35), quercetin enhanced the survival rate of damaged PC12 cells, promoted cell proliferation, counteracted Aβ toxicity, and exerted robust neuroprotective effects (Yu et al., 2020).

## 5 Quercetin exerts antidepressant effects through neuroprotection

Neural circuits serve as the essential communication pathways between neurons, relying on efficient neural information transmission to maintain normal function. Disruptions in this information flow can impair neural circuit integrity, and developmental abnormalities within the nervous system often contribute to the onset of mental disorders (Wu et al., 2018). In a chronic unpredictable mild stress (CUMS) mouse model, quercetin effectively attenuated CUMS-induced depression-like behaviors. Mechanistic investigations revealed that quercetin modulated the expression of neural markers in the hippocampal dentate gyrus (DG), including Forkhead Box G1 (FOXG1), cAMP-response element binding protein (CREB), and brain-derived neurotrophic factor (BDNF) (Ma et al., 2021).

In a chronic social defeat stress (CSDS) mouse model, quercetin increased the frequency of spontaneous excitatory postsynaptic currents (SEPSCs) and spontaneous inhibitory postsynaptic currents (SIPSCs) in the medial prefrontal cortex (MPFC) and hippocampus. Moreover, it suppressed the stress-induced activation of microglia and astrocytes in these brain regions, whose activation had previously decreased SEPSC and SIPSC frequencies. By enhancing neuronal activity in the MPFC and hippocampus, quercetin significantly alleviated depressive behaviors, highlighting its antidepressant effects mediated by neuroprotective activity, with astrocyte regulation playing a pivotal role (Zhang et al., 2020a).

Rinwa demonstrated in an OB model that OB mice exhibited hyperactivation of the HPA axis and elevated serum corticosterone (CORT) levels. Quercetin treatment lowered CORT levels, inhibited CRF induced anxiety and depression like responses, and suppressed microglia-mediated neuroinflammatory and neurotoxic reactions. By restoring neuronal integrity in the cerebral cortex and hippocampus of OB mice, quercetin exerted robust neuroprotective effects, thereby contributing to its antidepressant properties (Rinwa and Kumar, 2013).

Estrogen receptors, including estrogen receptor α (ERα) and estrogen receptor β (ERβ), play crucial roles in depression (Zhang et al., 2024) and cardiovascular disease (Aryan et al., 2020). The effects of estrogen are dependent on its binding to Erα and/or Erβ in various tissues including the brain, thereby promoting behaviors such as cognition and emotion (Fuentes and Silveyra, 2019). Behavioral tests for anxiety, depression, and memory were conducted on ovariectomized female mice. Through the investigation of ER levels in six brain regions, it was found that the mice exhibited anxiety-like and depression-like behaviors, as well as memory impairments (Baek et al., 2024). In an LPS-induced depression model, behavioral analysis of mice revealed that the high susceptibility of aged female mice to external factors leading to depression might be related to changes in estradiol (E2) hormone levels. This suggests that E2 has a protective effect on depressed mice, further indicating that ERα plays a more significant role than ERβ in the protection conferred by E2 in LPS-treated aged female mice, furthermore, knocking down ERα, rather than ERβ, was found to ameliorate LPS-induced apoptosis in PC12 cells (Jiang et al., 2021). BDNF, a key protein involved in depression, anxiety, and cardiovascular disorders, has been extensively studied. Quercetin treatment significantly upregulated BDNF protein expression and enhanced the phosphorylation of downstream targets, including TrkB, Akt, and ERK1/2. These findings suggest that quercetin exerts antidepressant and cardioprotective effects primarily through the BDNF-mediated PI3K/Akt and MAPK/ERK signaling pathways (Sun et al., 2021).

## 6 Quercetin suppresses proinflammatory factors and immune cell activation to exhibit antidepressant effects

Inflammation has emerged as a pivotal etiological factor in depression, with mounting evidence highlighting its intricate link to both anxiety and depressive disorders. Stress-induced neuroinflammation is recognized as a key pathological mechanism underlying the development of mental illnesses, including depression and anxiety (Wrobel-Biedrawa and Podolak, 2024). By suppressing proinflammatory cytokines, depressive symptoms can be alleviated, suggesting that anti-inflammatory interventions may represent a promising avenue for personalized treatment strategies, potentially augmenting the efficacy of conventional antidepressants. Research into anti-inflammatory compounds and their antidepressant mechanisms holds significant promise for developing novel therapeutic agents.

Inflammation encompasses two primary forms: peripheral and central inflammation. Peripheral inflammation stimulates the immune system to produce proinflammatory cytokines, whereas central inflammation, or neuroinflammation, involves immune responses within the central nervous system. Among the proinflammatory cytokines associated with depression, IL-6, IL-1β, and TNF-α are most frequently studied, while interleukin-10 (IL-10) acts as a crucial anti-inflammatory factor. Additionally, the central nervous system harbors immune cells such as microglia and astrocytes, which play integral roles in neuroinflammatory processes (Kohler et al., 2016).

In the OB model, increased levels of inflammatory mediators IL-6 and TNF-α were observed, which were significantly reduced by quercetin administration. These findings indicate that neuroinflammation, particularly glial cell activation, may contribute to neurodegeneration, increased neuronal apoptosis, and impaired neural regeneration, suggesting that quercetin exerts antidepressant effects by modulating neuroinflammatory pathways (Rinwa and Kumar, 2013). In the CUMS model, quercetin’s antidepressant mechanism was closely associated with its anti-inflammatory properties. Histological analysis revealed elevated concentrations of proinflammatory factors, including IL-6, IL-1β and TNF-α, in the mouse brain tissue of the CUMS group, which were markedly decreased following a glycoside of quercetin treatment (Shen et al., 2019).

In a 1,2-dimethylhydrazine (DMH)-induced colorectal cancer mouse model, depressive-like behaviors were evident in open field and forced swim tests, accompanied by increased IL-1β and TNF-α expression and decreased BDNF levels. The combination of quercetin and exercise therapy significantly reduced immobility time, increased social interaction, decreased serum inflammatory cytokines, mitigated prefrontal cortex inflammation, and enhanced BDNF signaling, thereby exerting potent antidepressant effects (Sadighparvar et al., 2020). Similarly, in a LPS-induced depressive-like model, administration of 10 mg/kg quercetin restored sucrose preference, reduced immobility in the tail suspension and forced swim tests, and decreased serum levels of proinflammatory cytokines IL-1β and TNF-α (Sah et al., 2011).

## 7 Mechanisms of quercetin's antidepressant and anxiolytic effects

Previous research has established that stress, 5-HT deficiency, and HPA axis dysregulation are key contributors to the development of anxiety and depression in both humans and animals. The therapeutic targets of quercetin for anxiety and depression are multi-faceted, encompassing the HPA axis, neurotransmitter systems, and BDNF, which underscore its multi-targeted and multi-modal treatment profile. Its sites of action, including the cerebral cortex, hippocampus, and hypothalamus, highlight the intricate mechanisms through which quercetin exerts its therapeutic effects on mood disorders. These findings emphasize the importance of further mechanistic studies to fully elucidate quercetin’s role in treating anxiety and depression. Notably, recent investigations (Zhang et al., 2020b) in zebrafish models (*Danio rerio*) have shown that moderate doses of quercetin can improve the fish’s health status and effectively alleviate behavioral phenotypes associated with schooling abnormalities and anxiety-like behaviors. Conversely, high concentrations (1,000 μg/L) of quercetin exert detrimental effects on these behaviors. These results suggest that low concentrations of quercetin confer anxiolytic protection in zebrafish, whereas high concentrations may exacerbate anxiety-like symptoms. However, whether similar concentration-dependent effects of quercetin exist in murine models remains an open question, warranting further investigation.

## 8 Prospects and outlook for the application of quercetin

Currently, various quercetin-based products targeting lung health are commercially available, yet no equivalent formulations exist for neurological disorders. Accumulating evidence suggests that quercetin, a naturally occurring flavonoid, exhibits promising anxiolytic and antidepressant properties. Mechanistically, quercetin modulates neurotransmitter systems, including 5-HT, GABA, and Glu, thereby exerting its anti-anxiety effects. These findings imply that dietary supplementation with quercetin-rich foods may represent a viable strategy for mitigating stress - induced anxiety.

From a clinical perspective, the etiology of anxiety disorders is intricately linked to endocrine dysregulation. Existing studies have demonstrated that within the context of the endocrine hypothesis, quercetin can ameliorate anxiety symptoms by modulating the HPA axis. However, the potential role of quercetin in improving anxiety via the hypothalamic - pituitary - gonadal (HPG) and hypothalamic - pituitary - thyroid (HPT) axes remains unelucidated, presenting an important avenue for future research. Additionally, although anxiety disorders are associated with immune system dysfunctions, investigations into quercetin’s immunomodulatory-mediated anxiolytic effects remain limited, highlighting another critical research gap. As our understanding of anxiety disorders deepens, quercetin’s anti-anxiety mechanisms are expected to attract increasing attention, providing a theoretical basis for the development of novel anxiolytic medications.

In contrast to single-mechanism drugs, quercetin simultaneously targets multiple pathological pathways underlying anxiety and depression, including neurotransmitter dysregulation, immune activation, oxidative stress, and neural circuit dysfunction. This multi-target property renders quercetin particularly suitable for complex comorbid conditions. As a natural dietary constituent with favorable safety and tolerability profiles, quercetin can be readily incorporated into daily diets, offering a distinct advantage over synthetic pharmaceuticals (Cui et al., 2022). Nevertheless, challenges remain, such as enhancing its bioavailability and expanding preclinical research across diverse animal models.

Despite the need for many clinical trials to validate its efficacy, quercetin’s natural origin, low toxicity, and multi-target actions hold great promise for the prevention, adjuvant treatment, and combination therapy of mental disorders. Future research efforts should prioritize elucidating precise molecular mechanisms, innovating drug delivery systems, and clinical study designs. These endeavors are crucial for transforming quercetin from a dietary supplement into a precision anti-psychiatric medication, thereby providing innovative solutions to the global mental health crisis.
